# P2Y2R Deficiency Ameliorates Hepatic Steatosis by Reducing Lipogenesis and Enhancing Fatty Acid β-Oxidation through AMPK and PGC-1α Induction in High-Fat Diet-Fed Mice

**DOI:** 10.3390/ijms22115528

**Published:** 2021-05-24

**Authors:** Theodomir Dusabimana, Eun Jung Park, Jihyun Je, Kyuho Jeong, Seung Pil Yun, Hye Jung Kim, Hwajin Kim, Sang Won Park

**Affiliations:** 1 Department of Pharmacology, Institute of Health Sciences, Gyeongsang National University College of Medicine, Jinju 52727, Korea; odomy2020@gmail.com (T.D.); foreverpak1@nate.com (E.J.P.); jeri1984@naver.com (J.J.); khjeong@gnu.ac.kr (K.J.); spyun@gnu.ac.kr (S.P.Y.); hyejungkim@gnu.ac.kr (H.J.K.); 2 Department of Convergence Medical Sciences, Institute of Health Sciences, Gyeongsang National University Graduate School, Jinju 52727, Korea

**Keywords:** P2Y2R, AMPK, fatty acid β-oxidation, hepatic steatosis, NAFLD

## Abstract

Non-alcoholic fatty liver disease (NAFLD) is a chronic metabolic liver disease associated with obesity and insulin resistance. Activation of the purinergic receptor P2Y2R has been reported to promote adipogenesis, inflammation and dyslipidemia in adipose tissues in obese mice. However, the role of P2Y2R and its mechanisms in NAFLD remain unknown. We hypothesized that P2Y2R deficiency may play a protective role in NAFLD by modulating lipid metabolism in the liver. In this study, we fed wild type and P2Y2R knockout mice with a high-fat diet (HFD) for 12 weeks and analyzed metabolic phenotypes. First, P2Y2R deficiency effectively improved insulin resistance with a reduction in body weight and plasma insulin. Second, P2Y2R deficiency attenuated hepatic lipid accumulation and injury with reduced alanine aminotransferase (ALT) and aspartate aminotransferase (AST) levels. Third, P2Y2R deficiency decreased the expression of fatty acid synthesis mediators (cluster of differentiation (CD36), fatty acid synthase (FAS), and stearoyl-CoA desaturase 1 (SCD1)); and increased the expression of adipose triglyceride lipase (ATGL), a lipolytic enzyme. Mechanistically, P2Y2R deficiency increased the AMP-activated protein kinase (AMPK) activity to improve mitochondrial fatty acid β-oxidation (FAO) by regulating acetyl-CoA carboxylase (ACC) and carnitine palmitoyltransferase 1A (CPT1A)-mediated FAO pathway. In addition, P2Y2R deficiency increased peroxisome proliferator-activated gamma co-activator-1α (PGC-1α)-mediated mitochondrial biogenesis. Conclusively, P2Y2R deficiency ameliorated HFD-induced hepatic steatosis by enhancing FAO through AMPK signaling and PGC-1α pathway, suggesting P2Y2R as a promising therapeutic target for NAFLD.

## 1. Introduction

Non-alcoholic fatty liver disease (NAFLD) is one of the most common chronic liver diseases and associated with metabolic disorders such as obesity, hypertension, dyslipidemia and type 2 diabetes (T2D) [1]. NAFLD is characterized by excessive fat accumulation in the liver, known as hepatic steatosis, affecting 25% of global population. NAFLD can progress to non-alcoholic steatohepatitis (NASH), cirrhosis, liver failure and hepatocellular carcinoma [2,3,4]. Accumulating evidence indicates that hepatic steatosis is common in individuals with insulin resistance and obesity [5]. Insulin resistance is a key pathogenic feature of NAFLD, substantially contributing to the development of steatosis through lipogenesis or lipid oversupply to the liver [6]. Therefore, the design of novel therapeutic drugs that enhance fat burning (β-oxidation) in the liver makes a significant contribution for alleviating NAFLD and obesity-related metabolic disorders.

Several metabolic adaptation processes during the development of NAFLD are induced for the prevention of excessive fat accumulation, such as increased mitochondrial fatty acid β-oxidation (FAO). However, mitochondrial respiratory chain (MRC) activity is concomitantly reduced or impaired in NAFLD, further aggravating mitochondrial dysfunction and oxidative stress. Thus, therapeutic drugs that improve both FAO and MRC activity, and mitochondrial antioxidants such as glutathione, could effectively alleviate hepatic steatosis and oxidative damage of NAFLD [7]. Importantly, hepatic lipogenesis and FAO are tightly regulated by malonyl-CoA which is the first intermediate substrate produced by acetyl-CoA carboxylase (ACC) for lipogenesis. Malonyl-CoA is also an allosteric inhibitor of carnitine palmitoyltransferase 1A (CPT1A) which is a rate-limiting step of carnitine shuttle for FAO and the gene expression and enzymatic activity of CPT1A are regulated by dietary, metabolic and epigenetic factors [8]. Thus, malonyl-CoA can be an important therapeutic point for regulating lipid metabolism by acting as the first substrate for lipogenesis and an FAO inhibitor [9]. Recent studies have reported that AMP-activated protein kinase (AMPK), a metabolic sensor of energy homeostasis, plays an important role in regulating lipid metabolism, thermogenesis and the development of adipose tissues [10]. AMPK activation inhibits lipogenesis by phosphorylation of ACC1 at Ser79, decreasing malonyl-CoA levels and promotes FAO by relieving the inhibition of CPT1A [11,12]. Liver-specific activation of AMPK reduces hepatic steatosis and inflammation due to promotion of FAO and inhibition of lipogenesis in mouse animal models of NAFLD [12,13]. AMPK also indirectly regulates FAO by controlling mitochondrial biogenesis through peroxisome proliferator-activated gamma co-activator-1α (PGC-1α) [14]. PGC-1α has shown to increase expression of genes important to gluconeogenesis, FAO, lipid transport and mitochondrial biogenesis processes [15,16]. In a mouse model of hepatic steatosis, the PGC-1α activity was impaired, and genes involved in mitochondrial biogenesis and FAO including Cpt1a were suppressed [17,18], suggesting a therapeutic benefit for PGC-1α activation. Taken together, balanced FAO and MRC activity and enhanced FAO and PGC-1α activity through AMPK activation may be promising therapeutic targets for ameliorating obesity-induced NAFLD [10,12].

Nucleotides can be released extracellularly in the form of ATP or UTP, to act as physiological second messengers or as danger signals in pathophysiological situations [19]. These extracellular nucleotides stimulate the purinergic P2 (P2Y) receptor, which consists of eight subtypes (P2Y1, P2Y2, P2Y4, P2Y6, and P2Y11-14) [19,20]. Particularly, P2Y2R is expressed in the most metabolic organs associated with obesity and insulin resistance including liver, adipose tissues, skeletal muscle, kidney, intestines and pancreas [20,21,22]. Activation of P2Y2R mediates several cellular pathways; in regenerating livers, P2Y2R induces hepatocyte proliferation and cell cycle progression through extracellular signal- regulated kinase (ERK) signaling and the downstream transcriptional activation [23]. Previously, it has been reported that constitutive P2Y2R activation inhibits basal lipolysis, whereas pharmacological antagonism or knockdown of P2Y2R increases intracellular cAMP levels and enhances basal lipolysis in human adipocytes [24]. P2Y2R also plays an active role in adipogenesis and expansion of adipose tissue, lipogenesis and inflammation in white adipose tissue, thereby promoting HFD-induced obesity and insulin resistance [21,22,25]. Taken together, the role of P2Y2R in the pathogenesis of NAFLD remains unknown despite its strong relationship with obesity-induced dyslipidemia. Here, we investigated whether P2Y2R deficiency plays a protective role focused on the lipogenesis and FAO signaling pathway in high-fat diet (HFD)-fed mice.

## 2. Results

### 2.1. P2Y2R Deficiency Reduced Insulin Resistance in HFD-Fed Mice

**Figure 1:**
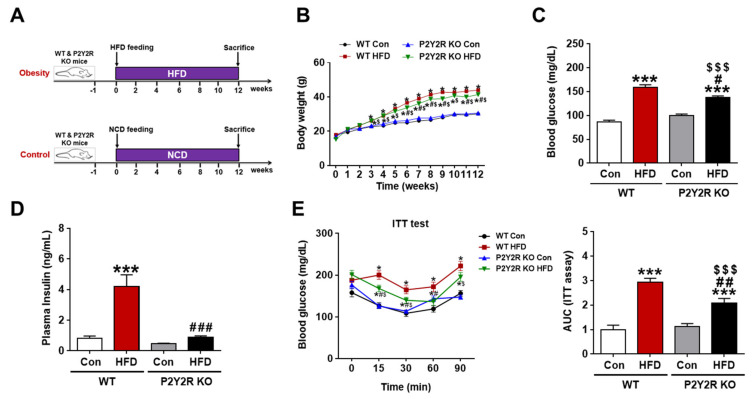
P2Y2R deficiency reduces insulin resistance in HFD-fed mice. (**A**) Experimental scheme for generating HFD-induced hepatic steatosis, where WT and P2Y2R KO mice were fed with a normal chow diet (NCD) or HFD for 12 weeks. (**B**) Weekly changes in the body weight in WT and KO mice for 12 weeks of feeding (*n* = 9) (**C**) Blood glucose levels were determined in WT or KO mice after overnight (15 h) fasting (*n* = 9). (**D**) Plasma insulin levels were measured after sacrifice in mice fed for 12 weeks (*n* = 8). (**E**) Insulin tolerance test (ITT) were performed in WT or KO mice fed for 12 weeks, and the corresponding areas under the curves (AUC) were calculated (*n* = 9). Data are presented as the mean ± SEM. One-way ANOVA was used for statistical analysis followed by Bonferroni’s multiple comparison test. * *p* < 0.05, *** *p* < 0.001 vs. WT control mice; and ^#^ *p* < 0.05, ^##^ *p* < 0.01, ^###^ *p* < 0.001 vs. WT HFD mice; and ^$^ *p* < 0.05, ^$$$^ *p* < 0.001 vs. KO control mice.

To determine the role of P2Y2R in the pathogenesis of HFD-induced NAFLD, wild-type (WT) and P2Y2R knockout (KO) mice were fed with normal chow diet (NCD) or HFD for 12 weeks (Figure 1A). The mRNA expression of P2Y2R were slightly increased in HFD-fed mice with no significance, and no P2Y2R expression was confirmed in KO mice (Supplementary Figure S1A). We monitored changes in body weights weekly and found that KO mice exhibited a significant decrease in body weights compared to WT mice from 6 weeks of HFD feeding (Figure 1B). First, we measured the levels of fasting blood glucose and plasma insulin. HFD feeding significantly induced blood glucose levels, but KO mice showed lower induction than WT mice (Figure 1C). Plasma insulin levels of HFD-fed KO mice were significantly reduced to the levels similar to the NCD-fed control mice (Figure 1D). Second, we performed insulin tolerance tests to determine the effect of P2Y2R deficiency on insulin resistance. HFD-fed KO mice significantly increased insulin sensitivity compared to WT after insulin injection as indicated by area under the curve (AUC) analysis (Figure 1E). In addition, since the adipose tissue expansion in obesity is associated with impaired adipogenesis and inflammation-induced insulin resistance [26,27], we examined the morphology of white adipose tissue in WT and KO mice (Supplementary Figure S1B). HFD feeding significantly increased the adipocyte size; however, P2Y2R deficiency reduced the adipocyte size and apoptosis, which significantly reduced crown-like structures. These results indicate that P2Y2R deficiency improves insulin resistance in HFD-fed mice.

### 2.2. P2Y2R Deficiency Attenuated Hepatic Steatosis and Cellular Injury in HFD-Fed Mice

**Figure 2:**
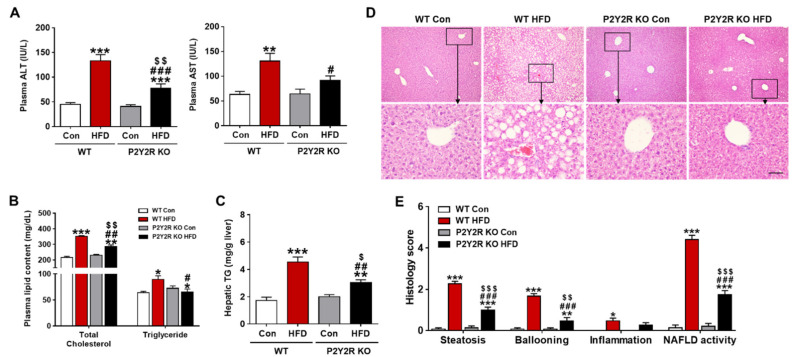
P2Y2R deficiency attenuates hepatic steatosis and cellular injury in HFD-fed mice. (**A**) Plasma ALT and AST levels were measured to assess hepatic injury (*n* = 8). (**B**,**C**) Plasma total cholesterol and triglyceride (*n* = 8), and hepatic triglyceride levels were determined to assess lipid accumulation (*n* = 5). (**D**) Representative images of H&E staining from liver sections were presented for evaluating hepatic steatosis and pathological changes (*n* = 5). (**E**) Hepatic steatosis, ballooning, and lobular inflammation were semi-quantitatively evaluated from H&E-stained sections to determine NAFLD activity score (NAS) as detailed in materials and methods. Data are presented as the mean ± SEM. One-way ANOVA was used for statistical analysis followed by Bonferroni’s multiple comparison test. * *p* < 0.05, ** *p* < 0.01, *** *p* < 0.001 vs. WT control mice; and ^#^ *p* < 0.05, ^##^ *p* < 0.01, ^###^ *p* vs. WT HFD mice; and ^$^ *p* < 0.05, ^$$^ *p* < 0.01, ^$$$^ *p* < 0.001 vs. KO control mice. Scale bar, 100 µm.

Long-term HFD intake causes excessive lipid accumulation in the liver, leading to hepatic steatosis and dysfunction in patients with NAFLD [28]. To determine the role of P2Y2R on hepatocellular injury, we measured plasma alanine aminotransferase (ALT) and aspartate aminotransferase (AST). The levels were significantly increased in WT HFD mice, compared to control; however, the levels were attenuated in KO HFD mice (Figure 2A). In addition, the plasma lipid content was assessed by total cholesterol and triglyceride levels, which were also significantly reduced in KO HFD mice (Figure 2B). Then, we measured the content of hepatic triglyceride which is the major form of fat storage in hepatocytes [1]. The levels were significantly increased in WT HFD mice, but the levels were reduced in KO HFD mice (Figure 2C). Consistently, histological analysis of the liver revealed an increased lipid accumulation in WT HFD mice, while it was significantly reduced in the liver of KO HFD mice, as presented by histology scores (Figure 2D,E). Steatosis and hepatocyte ballooning were significantly increased in WT HFD mice, but attenuated in KO HFD mice. Lobular inflammation, which was rare in all samples, showed no significant differences in WT and KO mice fed with HFD. NAFLD activity score (NAS), the sum of steatosis, ballooning and inflammation scores, was significantly lower in KO HFD mice than WT HFD mice (Figure 2E). These data indicate that P2Y2R deficiency attenuates hepatic steatosis and cellular injury in HFD-fed mice.

### 2.3. P2Y2R Deficiency Decreased De Novo Lipogenesis in HFD-Fed Mice

**Figure 3:**
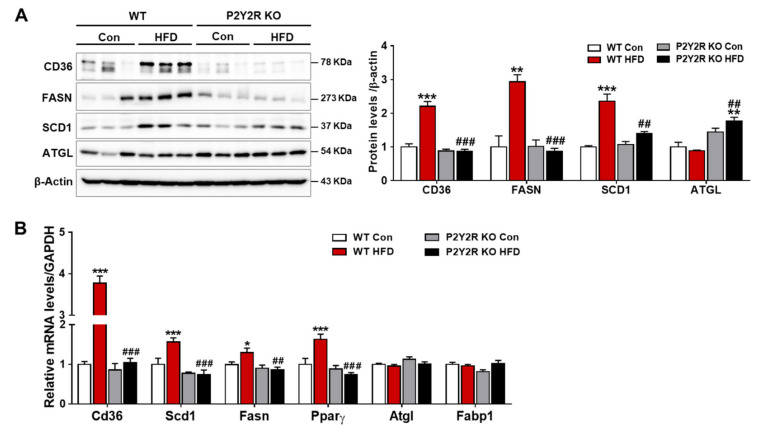
P2Y2R deficiency decreases de novo lipogenesis in HFD-fed mice. (**A**) Liver tissues were lysed to perform western blot analysis and the levels of proteins regulating lipid metabolism (CD36, FAS, SCD1 and ATGL) and β-actin (a loading control), were examined. Quantitative analysis of each protein was shown (*n* = 5). (**B**) The expression of genes involved in lipogenesis and lipolysis was determined by real-time PCR analysis. Relative mRNA levels were normalized to those of GAPDH (*n* = 3–5). Data are presented as the mean ± SEM. One-way ANOVA was used for statistical analysis followed by Bonferroni’s multiple comparison test. * *p* < 0.05, ** *p* < 0.01, *** *p* < 0.001 vs. WT control mice; and ^##^ *p* < 0.01, ^###^ *p* < 0.001 vs. WT HFD mice.

Hepatic steatosis is a condition of excess lipid accumulation in the liver, where lipid uptake and de novo synthesis surpass lipid oxidation and export. In NAFLD, hepatic uptake and de novo lipogenesis are increased, while lipid oxidation is insufficient or impaired due to mitochondrial dysfunction [28,29]. To investigate whether P2Y2R deficiency affects hepatic uptake and lipogenesis in NAFLD, we examined the expression of regulatory proteins involved in HFD-fed mice. Compared to WT mice, KO HFD mice showed a significant decrease in the expression of cluster of differentiation 36 (CD36), a translocase protein facilitating lipid transport, and the expression of fatty acid synthase (FAS) and stearoyl-CoA desaturase 1 (SCD1), enzymes for de novo lipogenesis (Figure 3A). In addition, KO HFD mice showed an increased expression of ATGL (adipose triglyceride lipase), a major lipase in liver as well as in adipose tissues, suggesting a regulatory role of P2Y2R in lipolysis. Consistently, real-time PCR analysis showed that KO HFD mice exhibited a significant reduction compared to WT HFD in the expression of Cd36, Scd1, Fasn, and Pparγ (peroxisome proliferator-activated receptor γ), genes regulating the lipid uptake and synthesis (Figure 3B). However, the gene expression of Atgl and Fabp1 showed no difference in WT and KO mice fed with HFD. These results indicate that P2Y2R deficiency attenuates hepatic steatosis in HFD-fed mice, suggesting a critical role of P2Y2R on hepatic lipogenesis and lipolysis in the development of NAFLD.

### 2.4. P2Y2R Deficiency Enhanced Mitochondrial Fatty Acid β-Oxidation in HFD-Fed Mice

**Figure 4:**
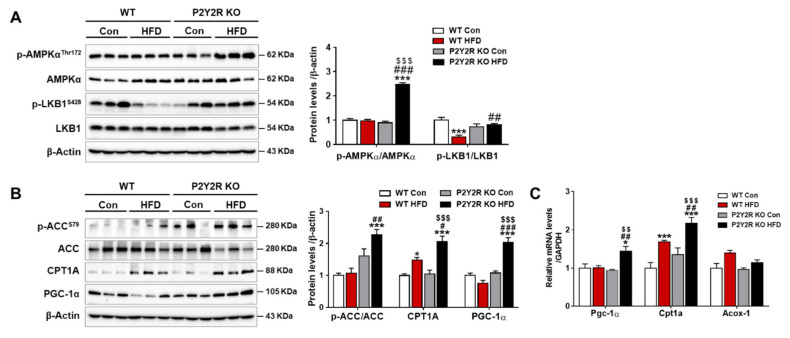
P2Y2R deficiency enhances mitochondrial fatty acid β-oxidation in HFD-fed mice. (**A**,**B**) Liver tissues were lysed to perform western blot analysis, and the protein levels of p-AMPK, p-LKB1, p-ACC, PGC-1α, CPT1A, and β-actin (a loading control), were examined. Quantitative analysis of each protein was shown (*n* = 5). (**C**) The mRNA expression of genes involved in fatty acid β-oxidation was determined by real-time PCR analysis. Relative mRNA levels were normalized to those of GAPDH (*n* = 3–5). Data are presented as the mean ± SEM. One-way ANOVA was used for statistical analysis followed by Bonferroni’s multiple comparison test. * *p* < 0.05, *** *p* < 0.001 vs. WT control mice; and ^#^ *p* < 0.05, ^##^ *p* < 0.01, ^###^ *p* < 0.001 vs. WT HFD mice; and ^$$^ *p* < 0.01, ^$$$^ *p* < 0.001 vs. KO control mice.

Despite conflicting studies of mitochondrial fatty acid β-oxidation (FAO), the impaired FAO in patients of NAFLD exacerbates mitochondrial dysfunction by promoting oxidative stress and inflammatory cytokines [29,30]. AMPK has shown to increase FAO by phosphorylating ACC1 and regulating PGC1-α activation [11]. To investigate a potential role of P2Y2R in the AMPK-mediated lipid metabolism, the phosphorylation of AMPK (Thr172) and its upstream kinase, liver kinase B1 (LKB1, Ser428), was determined in WT or KO mice after HFD feeding. The levels were significantly increased in KO mice compared to WT mice, reflecting that active LKB/AMPK signaling pathway was induced (Figure 4A). Consistently, the phosphorylation of ACC (Ser79) was increased in KO HFD mice compared to WT HFD mice (Figure 4B). P2Y2R deficiency also significantly increased the mRNA and protein levels of PGC1-α and CPT1A in HFD-fed mice, without changes in the mRNA levels of acyl-coA oxidase-1 (Acox-1), the first enzyme of peroxisomal β-oxidation (Figure 4B,C). These results indicate that P2Y2R deficiency inhibits hepatic steatosis by enhancing FAO through AMPK and PGC-1α activation.

### 2.5. P2Y2R Deficiency Improved Hepatic Mitochondrial Function in HFD-Fed Mice

**Figure 5:**
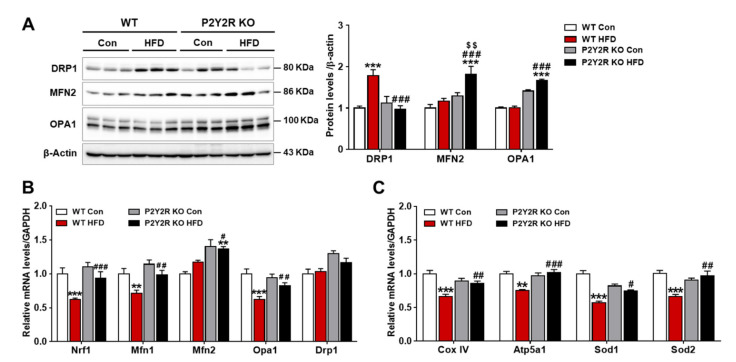
P2Y2R deficiency attenuates hepatic mitochondrial dysfunction in HFD-fed mice. (**A**) Liver tissues were lysed to perform western blot analysis, and the protein levels of DRP1, MFN2, OPA1 and β-actin (a loading control) were examined. Quantitative analysis of each protein was shown (*n* = 5). (**B**,**C**) The mRNA expression of genes involved in mitochondrial dynamics and respiratory and antioxidant functions was determined by real-time PCR analysis. Relative mRNA levels were normalized to those of GAPDH (*n* = 3–5). Data are presented as the mean ± SEM. One-way ANOVA was used for statistical analysis followed by Bonferroni’s multiple comparison test. ** *p* < 0.01, *** *p* < 0.001 vs. WT control mice; and ^#^ *p* < 0.05, ^##^ *p* < 0.01, ^###^ *p* < 0.001 vs. WT HFD mice; and ^$$^ *p* < 0.01 vs. KO control mice.

**Figure 6:**
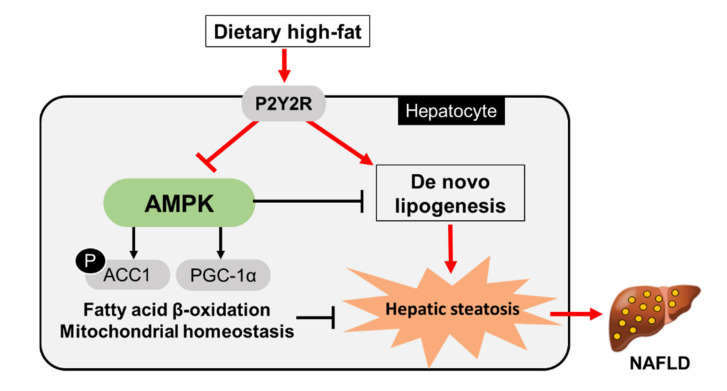
A schematic diagram illustrating the role of P2Y2R in HFD-induced hepatic steatosis. P2Y2R increases triglyceride accumulation and lipogenic gene expression, while inhibiting fatty acid β-oxidation and mitochondrial homeostasis through AMPK inhibition; thus, P2Y2R aggravates hepatic steatosis in HFD-induced obesity. In P2Y2R deficiency, AMPK activation enhances ATGL-mediated lipolysis and inhibits ACC activity, promoting CPT1A-mediated FAO and PGC-1α-mediated mitochondrial homeostasis, thereby ameliorating hepatic steatosis.

Given that PGC-1α, a key regulator of mitochondrial function, was induced in KO HFD mice, we investigated whether P2Y2R deficiency may restore mitochondrial dynamics and functions in NAFLD mouse model. Western blot analysis showed that the expression of protein regulating mitochondria fission (dynamin-related protein 1, (DRP1)) was significantly decreased in KO HFD mice compared to WT HFD mice; while the expression of mitofusin 2 (MFN2) and optic atrophy 1 (OPA1), which regulate mitochondria fusion, was upregulated in KO HFD mice compared to WT HFD mice (Figure 5A). Consistently, real-time PCR analysis showed that the expression of genes encoding mitochondrial biogenesis and dynamics (nuclear respiratory factor 1 (Nrf1), Mfn1, Mfn2, and Opa1) were significantly upregulated in KO HFD mice compared to WT HFD mice, without changing Drp1 mRNA expression (Figure 5B). Furthermore, the mRNA expression of genes for respiratory and antioxidant functions was analyzed; mitochondrial respiratory enzymes (complex IV (Cox IV) and ATP synthase mitochondrial F1 complex alpha subunit 1 (Atp5a1)) and antioxidant enzymes (superoxide dismutase 1 (Sod1) and Sod2) were upregulated in KO HFD mice compared to WT HFD mice (Figure 5C). These results suggest that P2Y2R deficiency may improve mitochondrial homeostasis and function through PGC-1α induction. In this study, HFD feeding has significantly increased the hepatic lipid accumulation; however, P2Y2R deficiency reduced lipogenesis and mitochondrial dysfunction by enhancing FAO and PGC-1α activity through AMPK signaling, suggesting P2Y2R as a promising therapeutic target for NAFLD (Figure 6).

## 3. Discussion

In this study, we investigated the role of P2Y2R in the development of hepatic steatosis and found the critical roles in hepatic lipid metabolism based on the following results. First, P2Y2R deficiency attenuated insulin resistance, hepatic triglyceride accumulation and hepatocellular injury in HFD-fed mice. Second, P2Y2R deficiency reduced hepatic lipogenesis, and increased lipolysis and mitochondrial FAO and biogenesis. Third, as a mechanism, P2Y2R deficiency improved hepatic steatosis through AMPK activation and PGC-1α induction. Therefore, we suggest P2Y2R as a promising therapeutic target for NAFLD.

NAFLD is strongly associated with T2D, as shown that ~70% of patients with T2D have NAFLD, where insulin resistance plays a critical role in hepatic dyslipidemia [31]. The liver is a major insulin-responsive organ that substantially contributes to the development of NAFLD by regulating hepatic fat accumulation, changes in energy metabolism, inflammatory signals and mitochondrial function [6]. Current treatment strategies aim to improve hepatic steatosis and inflammation by reducing insulin resistance and oxidative stress in obesity-induced NAFLD [5]. P2Y2R is widely expressed in metabolic organs, such as liver, white adipose tissue and skeletal muscle [20,21,22]. P2Y2R activation is involved in various hepatic cellular processes of proliferation, apoptosis, inflammation, and fibrosis [32,33,34]. A recent study has reported that P2Y2R promotes HFD-induced obesity and insulin resistance by stimulating adipogenesis and inflammatory cytokines, and altering adipokine production and lipid metabolism in adipose tissue [22]. Consistently, P2Y2R deficient mice inhibit excessive immune cell infiltration, reducing adipose tissue inflammation and ameliorating the HFD-induced metabolic phenotype [25]. In this study, we demonstrated that P2Y2R deficiency improved insulin resistance and hepatocellular injury in HFD-fed mice with a strong reduction in triglyceride accumulation, suggesting a critical role of P2Y2R in the development of NAFLD.

Adipose tissue dysfunction in obesity is caused by hypertrophic adipocytes and the macrophage infiltration. Hypertrophic adipocytes increases adipokines that have proinflammatory roles (e.g., IL-6, TNFα and MCP-1) [35]. Interestingly, the IL-6 and TNFα levels are significantly higher in adipose tissue than in liver, in patients with severe obesity, suggesting that adipose tissue is the main cytokine source in the progression of steatohepatitis [36]. In a previous study [22] and our current work demonstrated that P2Y2R promotes inflammation in adipose tissue; however, the liver inflammation was less scored and/or observed in both WT and KO mice fed with HFD (Figure 2E). The liver is an important target of adipose tissue, and fatty liver is due to an increase in circulating free fatty acids (FFAs) secondary to an increase in adipose tissue FFAs. Activation of adipose tissue macrophages also contributes to ectopic fat accumulation in the liver. Thus, treatments that restore adipose tissue insulin sensitivity and suppress inflammatory responses attenuate the pathology of NAFLD and inhibit its progression to the advanced stages [37,38]; P2Y2R is proposed as a promising therapeutic target.

NAFLD is characterized by excessive hepatic lipid accumulation, susceptible to oxidative stress and inflammation, and can progress to NASH, cirrhosis, liver failure and hepatocellular carcinoma [4,39]. Previous studies suggest that reducing hepatic lipid accumulation is an effective way to prevent the development of NAFLD [13,29]; thus, the precise mechanisms and molecular targets are being actively investigated. Our study demonstrated that genetic deletion of P2Y2R confers a significant resistance to HFD-induced obesity by decreasing hepatic steatosis, evidenced by a reduction in hepatic triglycerides and NAFLD activity scores. Hepatic lipids in patients with NAFLD come from dietary fatty acids (15%), de novo synthesis (30%) and free fatty acid influx from adipose tissue (60%) [30]. Circulating fatty acids are transported to hepatocytes via fatty acid translocase CD36, and their levels are elevated in patients with NAFLD. As a common target of lipogenic nuclear receptors, CD36 plays an important role in HFD-induced hepatic steatosis by modulating the rate of fatty acid uptake [40]. Hepatocyte-specific CD36 null-mice reduce hepatic lipid content and inflammatory markers, and improve insulin sensitivity [40,41]. Consistently, the present study demonstrated that P2Y2R deficiency reduced the expression of hepatic CD36 elevated in HFD-fed mice. P2Y2R deficiency also reduced the expression of lipogenic enzymes of FAS and SCD1, as well as their transcription factor PPARγ [42], while promoting a lipolytic enzyme, ATGL. This study strongly supports that P2Y2R plays an important role in hepatic steatosis in HFD-induced obesity and that targeting P2Y2R may ameliorate the development of NAFLD.

AMPK is a master regulator of metabolism and the liver-specific activation of AMPK has shown to protect against NAFLD by increasing fat oxidation and reducing hepatic lipogenesis, inflammation and fibrosis [11,13,43]. Pharmacological activation of AMPK has also shown to inhibit de novo lipid and cholesterol synthesis in rodent and primate preclinical models [44]. AMPK activation induces ACC phosphorylation that inhibits the conversion of acetyl-CoA to malonyl-CoA, a precursor in fatty acid synthesis and an allosteric inhibitor of CPT1A for fat oxidation. Here, we reported that AMPK was activated in KO mice fed with HFD, which increased the expression of phosphorylated ACC and CPT1A, thereby inhibits lipogenesis and promotes mitochondrial FAO. This is supported by an observation of increased fatty acid synthesis and reduced FAO in the liver of mice lacking AMPK phosphorylation sites on ACC1/ACC2 by knock-in mutations [45]. LKB1 is an upstream kinase activating AMPK activity by phosphorylation [46], and our previous study showed that the induction of LKB/AMPK pathway attenuates triglyceride levels and lipogenic proteins in hepatocytes [47]. Consistently, this study showed that the activation of LKB/AMPK was positively correlated with the suppression of lipogenesis in KO mice fed with HFD, suggesting a critical role of P2Y2R in hepatic steatosis through regulation of AMPK.

PGC-1α is a transcriptional cofactor, activating mitochondrial biogenesis and FAO and also controlling gluconeogenesis in the liver [48]. The PGC1α activity and mitochondrial homeostasis have been impaired in steatotic liver in a NAFLD mouse model [17], whereas PGC-1α overexpression results in increased hepatic FAO with reduced triacylglycerol accumulation and secretion in the rat liver [16]. AMPK in skeletal muscle directly phosphorylates PGC-1α for the active transcription of target genes of glucose uptake, FAO and mitochondrial biogenesis [14]. PGC-1α was also reported to regulate mitochondrial FAO and antioxidant enzymes in response to mitochondrial dysfunction and oxidative stress in NAFLD animal model [16,49]. In this regard, we investigated the role of P2Y2R on regulating PGC-1α in HFD-fed mice. We found that P2Y2R deficiency increased the expression of PGC-1α and restored the expression of genes regulating mitochondrial biogenesis and dynamics (Nrf1, Mfn2, Mfn1 and Opa1), and genes for respiratory (Cox IV and Atp5a1) and antioxidant enzymes (Sod1 and Sod2) altered in HFD-fed mice. Thus, we propose that P2Y2R has an important role in PGC-1α-mediated mitochondrial biogenesis and homeostatic function.

There are several limitations in the current study. First, studies using liver-specific P2Y2R KO mice are required to validate the direct role of P2Y2R on hepatic lipid metabolism, clarifying its precise application for treating patients with NAFLD. Second, additional studies are required to validate mitochondrial morphology and function, such as electron microscopic examination, respiratory enzyme activities and/or redox homeostasis in the liver of KO HFD mice.

In summary, P2Y2R deficiency improved insulin resistance, triglyceride accumulation, and hepatocellular injury by reducing hepatic lipogenesis and promoting mitochondrial FAO, thereby ameliorating NAFLD phenotype. Mechanistically, by increasing the AMPK activity, P2Y2R deficiency can regulate de novo lipogenesis, CPT1A-mediated FAO, and PGC-1α-induced mitochondrial biogenesis. We propose that targeting P2Y2R is a promising therapeutic strategy for NAFLD and obesity-induced metabolic disorders.

## 4. Materials and Methods

### 4.1. Experimental Animals

WT C57BL/6 mice (6-week old) were purchased from Koatech Co. (Pyeongtaek, South Korea) and homozygous P2Y2R KO mice on C57BL/6 background (strain B6.129P2-P2ry2tm1Bhk/J) were obtained from Jackson Laboratory. All mice were maintained in the animal facility at Gyeongsang National University (GNU-171130-M0058). All animal experiments were approved by the Institutional Board of Animal Research at Gyeongsang National University, and performed according to the National Institutes of Health guidelines for laboratory animal care. Mice were housed with an alternating 12-h light/dark cycle and provided with water and standard chow ad libitum.

### 4.2. NAFLD Mouse Model

WT and P2Y2R KO male mice were habituated for 1 week and then randomly divided into 4 groups (*n* = 9 per group). Mice were fed with NCD or HFD (60 Kcal % fat; Research Diets, Inc., New Brunswick, NJ, USA) for 12 weeks. The mice were weighed weekly for 12 weeks, and fasting blood glucose levels were measured from the tail vein using an Accu-Check glucometer (Roche Diagnostics, Mannheim, Germany) before sacrifice. After 12 weeks of feeding, all mice were sacrificed and liver and white adipose tissues were collected. The median and left lobes were immediately resected and snap-frozen in liquid nitrogen for storage at −80 °C or fixed in 10% buffered formalin for further analysis. Blood was collected from an inferior vena cava using a heparinized syringe, then centrifuged at 3000× *g* for 20 min, and the supernatants were stored at −80 °C for biochemical analysis.

### 4.3. Biochemical Assays

Plasma ALT and AST activities were measured using a commercial assay kit (IVD Lab Co., Uiwang, Korea) and a spectrophotometer (Shimadzu UV-1800 spectrophotometer, Tokyo, Japan). Mouse plasma insulin levels were determined by a commercial assay kit from Crystal Chem (Chicago, IL, USA) according to the manufacturer’s instructions. Plasma total cholesterol and triglyceride levels were measured using commercial assay kits (Cayman Chemicals, Ann Arbor, MI, USA) according to the manufacturer’s instructions. To perform insulin tolerance test (ITT), mice were intraperitoneally injected with 0.75 U/kg insulin (Humulin-R, Eli Lilly, Indianapolis, IN, USA) and blood glucose levels were measured at indicated times after injection using Accu-Check glucometer (Roche Diagnostics). AUC were calculated as an index of glucose or insulin intolerance.

### 4.4. Hepatic Triglyceride Assay

Hepatic triglyceride content was measured using a triglyceride colorimetric assay kit (Cayman Chemicals, Ann Arbor, MI, USA) according to the manufacturer’s instructions. Briefly, liver tissues (40 mg) were homogenized in 200 µL of the NP40 substitute assay reagent (Cayman Chemicals) containing protease inhibitors (Thermo Fisher Scientific, Waltham, MA, USA), and centrifuged for 10 min at 10,000× *g* at 4 °C to collect the supernatants. The sample and enzyme mixture were incubated for 30 min at 37 °C and the absorbance was measured at 540 nm using a VersaMax microplate reader (Molecular devices, San Jose, CA, USA).

### 4.5. Histological Examination

The liver and adipose tissues were fixed in 10% formalin solution, embedded in paraffin and sectioned at 5 µm of thickness. The sections were stained with hematoxylin and eosin (H&E) according to the standard protocols for histological analysis and all staining images were captured using a CKX41 light microscope (Olympus, Tokyo, Japan). The NAFLD activity score (NAS), established by the Pathology Committee of the Non-alcoholic steatohepatitis (NASH) Clinical Research Network [50], was used for hepatic pathological scoring; 3 histological features (steatosis, lobular inflammation, and hepatocellular ballooning) were evaluated semi-quantitatively. Steatosis was scored (0–3); 0 = 0–5%, 1 = 6–33%, 2 = 34–66%, and 3 = 67–100% of hepatocytes containing fat droplets. Lobular inflammation was scored (0–3); 0 = no foci, 1 = more than 2 foci per 200× field, 2 = 2–4 foci per 200× field, and 3 = greater than 4 foci per 200× field. Hepatocyte ballooning was scored (0–2); 0 = none, 1 = few, and 2 = many/prominent ballooned cells. The final NAS is the unweighted sum of the three scores of steatosis, inflammation, and ballooning; NAS ≥ 5 are diagnosed as “NASH” and NAS < 3 as “not NASH”.

### 4.6. Western Blot Analysis

Liver tissues were homogenized in the ice-cold radio-immunoprecipitation assay (RIPA) buffer with protease inhibitors (Thermo Fisher Scientific), sonicated and incubated for 20 min on ice. After centrifugation, the supernatant was transferred to a clean tube, and the protein concentration was determined using a Pierce^TM^ BCA protein assay kit (Thermo Fisher Scientific). The protein lysates were separated using sodium dodecyl sulphate-polyacrylamide gel electrophoresis (SDS-PAGE), transferred to polyvinylidene difluoride (PVDF) membranes, and blocked with 5% of skim milk or 3% bovine serum albumin (BSA). The membranes were incubated with primary antibodies against AMPK, p-AMPK, ACC, p-ACC, ATGL, CD36, FASN, SCD1, LKB1, p-LKB1 (from cell signaling technology, Danvers, MA, USA); CPT1A, MFN2, PGC-1α (from Abcam, Cambridge, MA, USA); DRP1 (Santa Cruz Biotechnology, Dallas, TX, USA); OPA1 (BD Biosciences, Franklin Lakes, NJ, USA); and β-actin (Sigma, St. Louis, MO, USA) in the blocking buffer solution at 4 °C overnight. Next, the membranes were incubated with the appropriate horseradish peroxidase (HRP)-conjugated secondary antibodies (Bio-Rad, Hercules, CA, USA) at room temperature for 1 h and then visualized with the ECL substrates (Bio-Rad). The ChemiDoc XRS+ System (Bio-Rad) was used to evaluate the density of protein bands, and relative protein levels were quantified using Image Lab^TM^ software (Bio-Rad).

### 4.7. Quantitative Real-Time Polymerase Chain Reaction (PCR) Analysis

The total RNA was extracted with Trizol (Invitrogen, Carlsbad, CA, USA) and converted into cDNA using the RevertAid Reverse Transcription System (Thermo Fisher Scientific) according to the manufacturer’s protocols. Real-time PCR analysis was performed with a CFX Connect real-time PCR System using iQ SYBR Green Supermix (Bio-Rad). Real-time PCR analysis was performed with an initial denaturation at 94 °C for 5 min, and the cycling conditions were 45 cycles of 10 s at 95 °C, 10 s at 60 °C and 30 s at 72 °C. Relative mRNA levels were normalized to those of glyceraldehyde 3-phosphate dehydrogenase (GAPDH). The primer sequences are listed in Table 1.

### 4.8. Statistical Analysis

Statistical significance was determined using one-way analysis of variance (ANOVA), followed by Bonferroni’s multiple comparison test. All statistical analyses were performed with GraphPad Prism 7 Software v.7.00 (GraphPad Software Inc., La Jolla, CA, USA). Data were expressed as the mean ± SEM. * *p* < 0.05, ** *p* < 0.01, *** *p* < 0.001 vs. WT control mice; and ^#^ *p* < 0.05, ^##^ *p* < 0.01, ^###^ *p* < 0.001 vs. WT HFD mice; and ^$^ *p* < 0.05, ^$$^ *p* < 0.01, ^$$$^ *p* < 0.001 vs. KO control mice.

## Figures and Tables

**Table 1:** The primer sequences used for real-time PCR analysis in this study.

Gene	Forward Primers (5′–3′)	Reverse Primers (5′–3′)
Atgl	GGCCAACGCCACTCACATCTAC	CACGGATGGTCTTCACCAGGTTG
Cd36	CTGGGACCATTGGTGATGAAA	CACCACTCCAATCCCAAGTAAG
Fasn	AGTGGACGCACCTTAGAGGCAG	ACTTGCTGCACTTCTTGGACACG
Scd1	CAACTTCACCACGTTCTTCATC	CCCGTCTCCAGTTCTCTTAATC
Pparγ	CGGTGTGTATGAAGCCATCT	TAAGGAACTCGCGTGTGATAAA
Fabp1	GTACCAATTGCAGAGCCAGGAGA	GGTCCATAGGTGATGGTGAGTTTG
Pgc-1α	AGCCGTGACCACTGACAACGAG	GCTGCATGGTTCTGAGTGCTAAG
Cpt1a	GAAGTGTCGGCAGACCTATT	GTCCTCCTCTCTATATCCCTGTT
Acox-1	CGCACATCTTGGATGGTAGT	GGCTTCGAGTGAGGAAGTTATAG
Nrf1	GAGCACGGAGTGACCCAAAC	TGTACGTGGCTACATGGACCT
Mfn1	TACTGGACTCAGTAAACGTGGC	CTCCGTGACCTCCTTGATCT
Mfn2	ACCGTCAAGAAGGATAAGCGACAC	GTGTTCCTGTGGGTGTCTTCAAGG
Opa1	CCAAGAACGAGTTGGAGAAGATGC	CACGTCATTGCATTCCAGCTCAGA
P2y2r	GTGCTCTACTTCGTCACCACCAG	CCATAAGCACGTAACAGACCAGGA
Drp1	ACCAAAGTACCTGTAGGCGATC	CATGGCATCAGTACCCGCAT
Cox IV	CGCTGAGCCTGATTGGCAAGAGA	TGGCAGACAGCATCGTGACATGG
Atp5a1	CCATGCCTCTAACACTCGACTTCA	CGGGTTCCAAGTTCAGGGACATAC
Sod1	CAGGGAACCATCCACTTCGAGC	CTGCACTGGTACAGCCTTGTGTA
Sod2	CCACCGAGGAGAAGTACCACGAG	CTCCTTATTGAAGCCAAGCCAGCC
Gapdh	ATGACATCACAGGTGAGCTGAAGG	CTCAAACCAGCCTTTCAGAATGGC

## Data Availability

Not applicable.

## Supplementary Materials

The following are available online at https://www.mdpi.com/article/10.3390/ijms22115528/s1, Figure S1.
