# Supplementary material for: P2Y2R Deficiency Ameliorates Hepatic Steatosis by Reducing Lipogenesis and Enhancing Fatty Acid β-Oxidation through AMPK and PGC-1α Induction in High-Fat Diet-Fed Mice
Source: Int J Mol Sci. 2021 May 24;22(11):5528. doi: 10.3390/ijms22115528 (PMC8197197; doi:10.3390/ijms22115528)
Supplement: Supplementary file 1 [file ijms-22-05528-s001.zip › ijms-1164160-supplementary.pdf]

Supplementary Figure S1

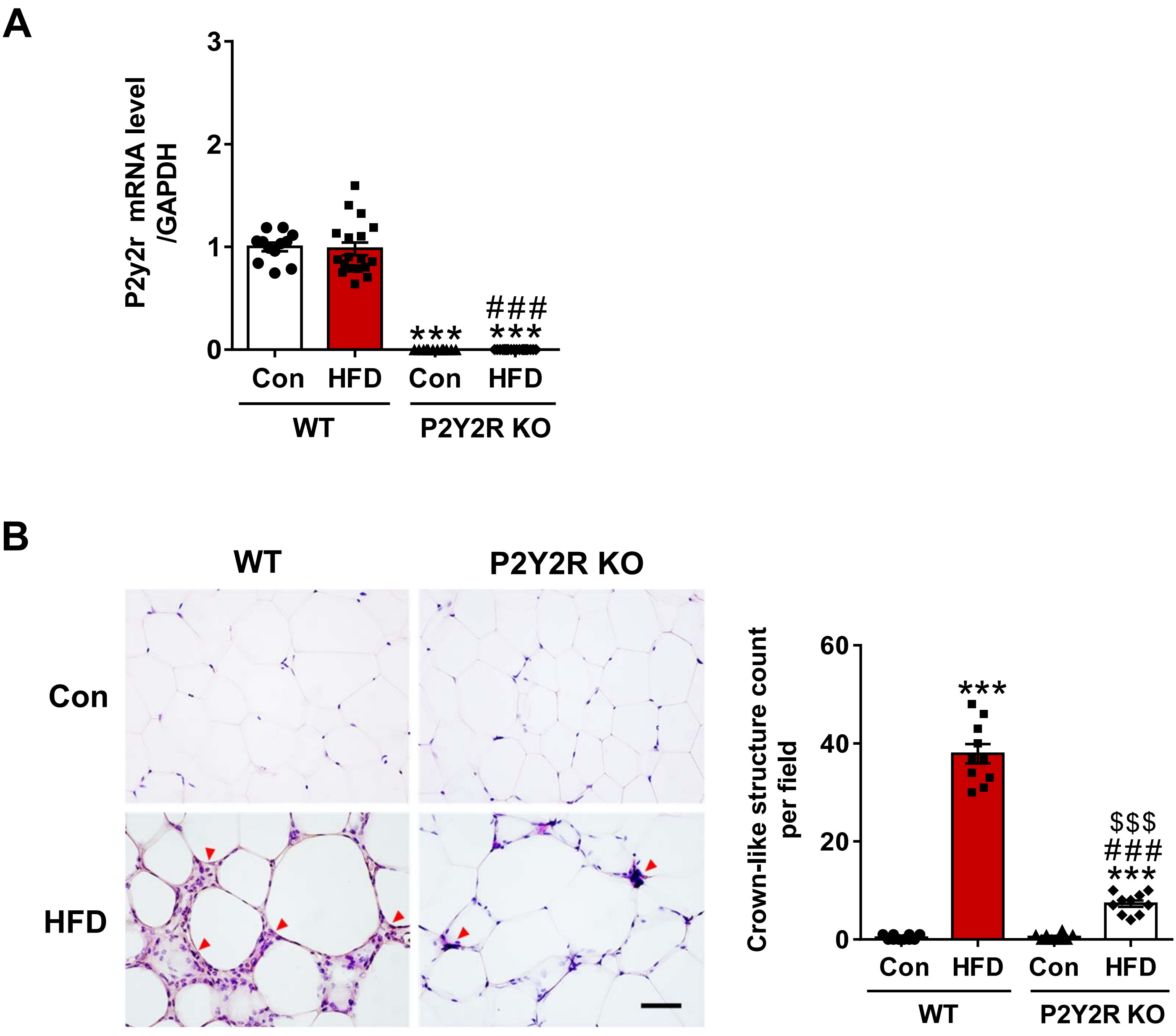

Figure S1. Hepatic P2Y2R mRNA expression and histological analysis of adipose tissue. (A) Relative mRNA expression of hepatic P2Y2R in WT and KO mice fed with NCD or HFD for 12 weeks; the mRNA levels were normalized to those of GAPDH (n = 3-5). (B) Representative images of white adipose tissue sections were presented after H&E staining from WT and KO mice fed with NCD or HFD for 12 weeks (n = 3). Red arrow heads indicate crown-like structures (CLS) formed in adipose tissue. Data are presented as the mean ± SEM. One-way ANOVA was used for statistical analysis followed by Bonferroni's multiple comparison test. ***p <0.001 vs WT control mice; and ###p <0.001 vs WT HFD mice; $^{\$\$\$}$p <0.001 vs KO control mice. Scale bar, 50 μm.
